# Correlation of Hypertension With Adverse Outcomes Following Total Hip Arthroplasty: A Matched Cohort Analysis

**DOI:** 10.5435/JAAOSGlobal-D-25-00048

**Published:** 2026-02-05

**Authors:** Ismail Ajjawi, Anshu Jonnalagadda, Jonathan N. Grauer

**Affiliations:** From the Department of Orthopaedics and Rehabilitation, Yale School of Medicine, New Haven, CT.

## Abstract

**Introduction:**

Total hip arthroplasty (THA) is a widely performed procedure. Despite its overall success, adverse outcomes have been associated with defined comorbidities. Hypertension (HTN), which affects a notable portion of the adult population, may be one such factor but has not been independently studied in this regard.

**Methods:**

THA patients were abstracted from the 2010 to 2022 M165 Ortho PearlDiver Mariner Database. Adult patients with versus without HTN were matched 1:1 based on age, sex, and Elixhauser Comorbidity Index. Ninety-day adverse outcomes and 5-year implant survival rates were compared for the matched cohorts with multivariable analysis, adjusting for multiple comparisons.

**Results:**

Of 851,605 THA patients identified, HTN was noted for 601,475 (70.6%). After matching, there were 148,946 patients with and the same number without HTN. Patients with HTN were at independently greater odds of most individual adverse outcomes assessed, any adverse events (odds ratio [OR] 2.18), serious adverse events (OR 2.19), minor adverse events (OR 2.26), and readmissions (OR 1.52) (*P* < 0.001 for each). Furthermore, the 5-year implant survival rate was mildly lower in HTN group (96.5% versus 97.5%, *P* < 0.001).

**Discussion:**

The current, large-cohort study identified HTN to be diagnosed for more than half of patients undergoing THA. The clear correlation of HTN with many adverse outcomes and mildly (but statistically) lower 5-year implant survival highlights the need for further consideration of this variable. Although there could be other confounding factors that may not have been fully controlled, those with HTN are clearly an “at-risk” group.

Total hip arthroplasty (THA) is a widely performed procedure.^1,2^ Despite its general success, with over 250,000 cases performed in the United States annually and growing,^3^ adverse outcomes have been associated with patient variables.^4–8^ Hypertension (HTN), which affects a notable portion of the adult population, may be one such factor but has not been independently studied in this regard.

The correlation of defined comorbidities with adverse outcomes following THA has been studied. These include, but are not limited to, age, obesity, drug/alcohol abuse, peripheral vascular diseases, diabetes, anemia, smoking, and chronic pulmonary disease. Such factors have been associated with increased rates of postoperative medical complications, higher rates of revision, and lower functional outcomes.

HTN is a common condition that affects more than 30% of the world's adult population and is associated with challenges in care.^9–11^ Among patients undergoing total joint arthroplasty, HTN is highly prevalent with studies reporting rates of 76% to 81.7%.^12,13^ With major cardiac and noncardiac surgeries, HTN during the perioperative and postoperative phases of surgery has been shown to corelate with elevated the risk of cardiovascular events, cerebrovascular complications, bleeding, and mortality.^14–16^

Nonetheless, few studies have investigated HTN's correlation with patient outcomes following total joint arthroplasty, and these studies have yielding mixed results. A retrospective, single-institution study by Ong et al^17^ found uncontrolled HTN not to be a reliable predictor of total joint arthroplasty (TJA) outcomes. However, another single-institution study by Ahmed et al^18^ found HTN to correlate with delayed wound healing following THA, potentially increasing infection risk.

Given the above considerations, further research is warranted to explore the relationship between HTN and THA. By leveraging a large, national database, HTN can be isolated as an independent variable to be assessed in this regard and may be helpful in the evolution of care pathways.

## Methods

### Study Cohorts

This study used the M165Ortho PearlDiver Mariner Patient Claims Database (PearlDiver Technologies) covering the years 2010 to 2022. This national, administrative data set is widely used in hip research.^18–22^ Our Institutional Review Board has deemed PearlDiver studies exempt from review because all data are output in a deidentified and aggregate form. Patients undergoing THA were identified based on Current Procedural Terminology (CPT) code 27130. Patients were excluded if they were younger than 50 years; had concurrent neoplasms, infections, trauma; or had less than 90 days of follow-up in the data set. International Classification of Diseases (ICD) 9 and 10 codes were then used to identify patients with HTN. In this study, HTN was identified using ICD coding, specifically ICD-9 codes 401.0 to 405.9 and ICD-10 codes I10 to I15.^17^ These codes represent essential, secondary, and unspecified HTN as documented in administrative claims. However, the PearlDiver database does not include clinical information needed to distinguish between HTN subtypes (e.g., primary versus secondary), stage (stage 1 or stage 2), or whether the condition was controlled or uncontrolled. As such, these important subclassifications could not be captured or incorporated into our analysis. A total of 851,605 patients who underwent THA were identified, of which HTN was noted for 601,475 (70.6%) and not noted for 250,130 (29.4%) (Table 1). Those with HTN were, on average, older (64.8+/09.5 years old versus 62.1 ± 11.1 years old), more men (45.2% versus 40.2%), and of greater Elixhauser Comorbidity Index (ECI) (7.3 ± 3.9 versus 2.6 ± 2.4) (*P* < 0.001 for each). To address the above cohort differences, a one-to-one matching process was performed between hypertensive and nonhypertensive patient groups, matched on age, sex, and the ECI (a measure of overall comorbidity).^23^ Once done, there were no longer differences in age, sex, or ECI (*P* = 1.00 for each) (Table 1). This matching approach allowed for the adjustment of concurrent medical conditions, ensuring that differences in outcomes could be more accurately attributed to HTN rather than other underlying health factors.

**Table 1:** Demographics of Total Hip Arthroplasty Patients Organized by Hypertension Status

Factors	Unmatched		*P*	Matched		*P*
	Nonhypertensive	Hypertensive		Nonhypertensive	Hypertensive	
Total (n)	250,130	601,475		148,946	148,946	
Age, mean ± SD	62.1 ± 11.1	64.8 ± 9.5	**<0.001**	63.6 ± 10.3	63.6 ± 10.3	1.000
50-54	25,437 (10.1%)	48,078 (7.9%)		13,589 (9.1%)	13,589 (9.1%)	
55-59	36,517 (14.6%)	80,727 (13.4%)		20,743 (13.9%)	20,743 (13.9%)	
60-64	42,816 (17.1%)	102,664 (17.0%)		25,929 (17.4%)	25,929 (17.4%)	
65-69	39,210 (15.7%)	108,692 (18.1%)		24,909 (16.7%)	24,909 (16.7%)	
70-74	47,035 (18.8%)	123,464 (20.5%)		29,171 (19.6%)	29,171 (19.6%)	
75-79	22,749 (9.1%)	91,103 (15.1%)		17,237 (11.5%)	17,237 (11.5%)	
≥80	36,366 (14.5%)	46,747 (7.7%)		17,368 (11.6%)	17,368 (11.6%)	
Sex			**<0.001**			1.000
Female	149,619 (59.8%)	329,587 (54.8%)		87,433 (58.7%)	87,433 (58.7%)	
Male	100,511 (40.2%)	271,888 (45.2%)		61,513 (41.3%)	61,513 (41.3%)	
ECI, mean ± SD	2.6 ± 2.4	7.3 ± 3.9	**<0.001**	3.8 ± 3.0	3.8 ± 3.0	1.000

Bold entries are statistically significant.

ECI = Elixhauser Comorbidity Index, THA = total hip arthroplasty

### Adverse Events and Outcome Metrics

Ninety-day adverse events were identified using ICD-9 and ICD-10 coding, with occurrences identified independently and in aggregate groups. This approach is consistent with previous literature studying adverse events following THA in PearlDiver.^24–27^ Serious adverse events (SAEs) were recorded if any of the following occurred: cardiac events (including myocardial infarction and cardiac arrest), sepsis, deep vein thrombosis (DVT), surgical site infection (SSI), or pulmonary embolism (PE). Minor adverse events (MAEs) were noted if any of the following occurred: acute kidney injury (AKI), pneumonia, urinary tract infection (UTI), wound dehiscence, hematoma, or transfusion. Any adverse event (AAE) was noted if there was the occurrence of either a SAE or MAE. In addition, readmissions, identified through the Pearldiver “ADMISSIONS” function, were tracked for 90 days following surgery. Five-year outcome metrics included implant survival from time of THA procedure (CPT-27130) until revision of THA, defined by the following CPT codes: CPT-27134, CPT-27137, and CPT-27138.

### Data Analyses

The demographics of patients who underwent THA, including age, sex, and the ECI, were compared between hypertensive and nonhypertensive patients. Differences in categorical variables, such as sex, were assessed using chi-squared tests, whereas Student *t*-test was employed to evaluate differences in numerical variables like ECI and age. This was done before and following matching with significance defined as *P* < 0.05. Adverse events were compared between the matched groups using univariable analysis, which was conducted with chi-squared tests. A multivariable analysis was then performed, controlling for age, sex, and ECI, to determine odds ratios (ORs) and 95% confidence intervals. Significance for both univariable and multivariable analyses was adjusted for multiple comparisons using the Bonferroni correction, with significance defined as *P* < 0.003. Implant survival until revision within 5 years postsurgery was compared between hypertensive and nonhypertensive patients using Kaplan-Meier survival analysis. Significance was defined between the groups with the log-rank test. All statistical analyses were performed using Pearldiver RSuite software (Pearldiver Technologies). Figures were created using GraphPad Prism 10 (GraphPad Software).

## Results

### Adverse Events

Univariate analysis of adverse events following THA is shown in Table 2. Patients with HTN demonstrated a significantly higher incidence of AAEs compared with their nonhypertensive counterparts, with rates of 28,931 cases (19.4%) versus 17,799 cases (11.9%), respectively (*P* < 0.001). Similarly, the occurrence of SAEs was elevated in hypertensive patients, with 10,499 cases (7.0%) versus 6,776 cases (4.5%) in nonhypertensive patients (*P* < 0.001). Specific complications that were of greater incidence were cardiac events, sepsis, DVT, SSI, and PE (*P* < 0.001 for each). Furthermore, MAEs were observed in 22,472 hypertensive patients (15.1%), in contrast to 13,560 nonhypertensive patients (9.1%, *P* < 0.001), with significant greater incidence for AKI, pneumonia, wound issues, hematoma, and transfusions (*P* < 0.001 for each).

**Table 2:** Univariate Comparison of Adverse Events Following Total Hip Arthroplasty Between Cohorts Organized by Hypertension Status

Factors	Nonhypertensive	Hypertensive	*P*
AAE	17,799 (11.9%)	28,931 (19.4%)	**<0.001**
SAE	6,776 (4.5%)	10,499 (7.0%)	**<0.001**
Cardiac	138 (0.1%)	347 (0.2%)	**<0.001**
Sepsis	192 (0.1%)	583 (0.4%)	**<0.001**
DVT	574 (0.4%)	1,222 (0.8%)	**<0.001**
PE	278 (0.2%)	782 (0.5%)	**<0.001**
SSI	400 (0.3%)	621 (0.4%)	**<0.001**
MAE	13,560 (9.1%)	22,472 (15.1%)	**<0.001**
AKI	2,095 (1.4%)	4,739 (3.2%)	**<0.001**
Pneumonia	2,142 (1.4%)	4,994 (3.3%)	**<0.001**
UTI	5,346 (3.6%)	12,416 (8.3%)	**<0.001**
Wound	1,039 (0.7%)	1,525 (1.0%)	**<0.001**
Hematoma	1,037 (0.7%)	842 (0.5%)	**<0.001**
Transfusion	4,532 (3.0%)	2,609 (1.7%)	**<0.001**
Readmission	6,704 (4.5%)	10,826 (7.2%)	**<0.001**

AAE = any adverse event, AKI = acute kidney injury, DVT = deep vein thrombosis, MAE = minor adverse event, PE = pulmonary embolism, SAE = serious adverse event, SSI = surgical site infection, UTI = pneumonia, urinary tract infection

Bold entries are statistically significant.

**Figure 1:**
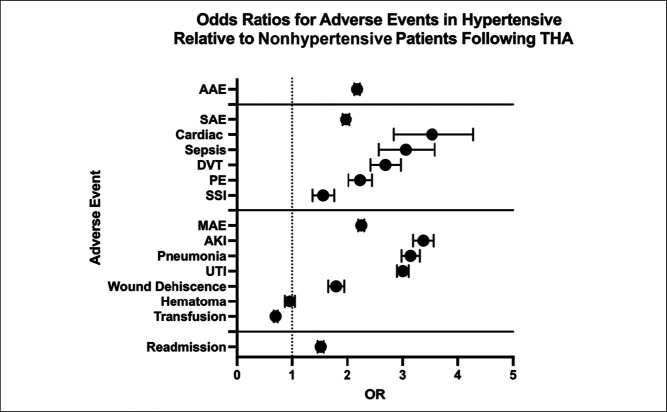
Forest plot demonstrating the multivariate comparison of adverse events in hypertensive versus nonhypertensive patients, controlling for age, sex, and ECI. Odds ratios (ORs) and 95% confidence intervals (CIs) are reported for each outcome. ECI = Elixhauser Comorbidity Index

Multivariate analysis then assessed for independent correlations (Table 3, Figure 1). Patients with HTN were at independently greater odds of aggregated AAE (OR 2.18), SAE (OR 2.19), MAE (OR 2.26) (*P* < 0.001 for each). In terms of individual adverse events, those with HTN were at greater odds of the following: cardiac (OR 3.48), AKI (OR 3.37), pneumonia (OR 3.14), sepsis (OR 3.03), UTI (OR 3.00), DVT (OR 2.68), PE (OR 2.23), wound (OR 1.79), and SSI (OR 1.55) (*P* < 0.001 for each). Furthermore, those with HTN were at greater odds of readmission (OR: 1.52, *P* < 0.001).

**Table 3:** Multivariate Comparison of Adverse Events Following Total Hip Arthroplasty in Hypertensive Relative to Nonhypertensive Patients: Reporting Odds Ratios and Confidence Intervals

Factors	Nonhypertensive	Hypertensive	*P*
AAE	Ref	**2.18 (2.13-2.23)**	**<0.001**
SAE	Ref	**1.97 (1.91-2.04)**	**<0.001**
Cardiac	Ref	**3.48 (2.84-4.28)**	**<0.001**
Sepsis	Ref	**3.03 (2.57-3.58)**	**<0.001**
DVT	Ref	**2.68 (2.42-2.97)**	**<0.001**
PE	Ref	**2.23 (2.02-2.45)**	**<0.001**
SSI	Ref	**1.55 (1.37-1.76)**	**<0.001**
MAE	Ref	**2.26 (2.20-2.31)**	**<0.001**
AKI	Ref	**3.37 (3.19-3.56)**	**<0.001**
Pneumonia	Ref	**3.14 (2.98-3.31)**	**<0.001**
UTI	Ref	**3.00 (2.90-3.11)**	**<0.001**
Wound	Ref	**1.79 (1.65-1.94)**	**<0.001**
Hematoma	Ref	0.96 (0.87-1.05)	0.440
Transfusion	Ref	**0.70 (0.67-0.74)**	**<0.001**
Readmission	Ref	**1.52 (1.46-1.57)**	**<0.001**

AAE = any adverse event, AKI = acute kidney injury, DVT = deep vein thrombosis, MAE = minor adverse event, PE = pulmonary embolism, SAE = serious adverse event, SSI = surgical site infection, UTI = pneumonia, urinary tract infection

Bold entries are statistically significant.

### Five-Year Survival Rate of Implants Before Revision

**Figure 2:**
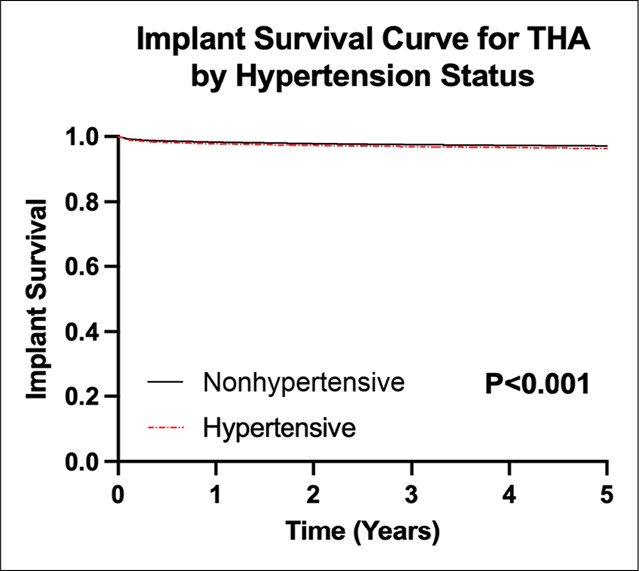
Kaplan-Meier survival curves showing implant survival at 5 years: Hypertensive (96.5% at 5 years) versus nonhypertensive (97.5% at 5 years) patients following total hip arthroplasty (Log-rank test, *P* < 0.001).

The rates of implant survival until revision were significantly different between the two groups (*P* < 0.001, Figure 2). At 5 years, the hypertensive group demonstrated a lower implant survival rate compared with the nonhypertensive group (96.5% versus 97.5%).

## Discussion

Previous studies assessing the potential correlation of HTN with THA outcomes have revealed mixed results.^17,18^ The current, large cohort comparison study found hypertensive THA patients to be at markedly greater odds of AAE (OR 2.18), SAE (OR 1.97), MAE (OR 2.26), readmissions (OR 1.52), and even slightly higher risk of 5-year revisions.

Specific complications such as cardiac events, AKI, sepsis, UTI, pneumonia, wound dehiscence, SSI, and DVT were particularly elevated in the hypertensive cohort, reinforcing the notion that HTN markedly exacerbates surgical risks. The increased odds of these complications are consistent with the prior literature.

HTN can increase the strain on the cardiovascular system,^28^ leading to a higher likelihood of perioperative cardiac events such as myocardial infarction or cardiac arrest. Elevated blood pressure also impairs renal function by causing damage to blood vessels in the kidneys,^29,30^ which may contribute to AKI following surgery. In addition, individuals with uncontrolled HTN often experience compromised immune function,^31,32^ which can elevate the risk of infections like sepsis, pneumonia, and SSIs, including wound dehiscence. Furthermore, HTN can contribute to the development of venous stasis and hypercoagulability,^33^ both of which are associated with a higher incidence of DVT. The need for further surgical intervention or extended hospital stays to manage these complications could explain the elevated risk of readmission and revision surgery in hypertensive patients. These compounded risks highlight how HTN, through its systemic effects, magnifies the potential for complications in the postoperative period, highlighting the necessity for thorough preoperative assessments, where healthcare providers should carefully evaluate the patient's cardiovascular status and any other comorbidities that may influence surgical outcomes.^34,35^

Given the high prevalence of HTN among surgical patients, particularly those undergoing major procedures like THA, it is vital to implement tailored management strategies. Previous studies have suggested notable benefits for achieving better blood pressure control, educating patients on lifestyle modifications, and collaborating with cardiology specialists for those at higher risk^36–38^ to avoid postoperative complications. In clinical practice, hypertensive patients may be considered “high risk” when they demonstrate features such as uncontrolled or severely elevated blood pressure, evidence of end-organ damage, resistant HTN, hypertensive heart disease, or multiple cardiovascular comorbidities. Although our data set does not allow identification of such subgroups, these characteristics typically prompt perioperative optimization or cardiology consultation before elective surgery. We have therefore clarified that our recommendation for consideration of cardiology involvement is based on standard clinical management principles rather than stratification available within the claims database. By proactively addressing HTN and its associated risks, healthcare providers can markedly enhance perioperative safety and reduce the likelihood of complications.

Future research should explore specific preoperative and postoperative strategies that could further optimize outcomes for this vulnerable patient group, potentially including the development of standardized protocols for managing hypertensive patients in the surgical setting.^39^ It is important to note that this study evaluates chronic, preexisting HTN as captured in administrative claims data and does not include intraoperative hemodynamic measurements. Therefore, the impact of short-term intraoperative blood pressure control on long-term outcomes, such as 5-year implant survival, cannot be assessed. Recommendations for standardized protocols should be interpreted as strategies for preoperative optimization of chronic HTN and mitigation of perioperative cardiovascular risk, rather than management of transient intraoperative blood pressure fluctuations. By enhancing our understanding of how HTN affects surgical outcomes, we can work toward improving the quality of care and long-term success rates for patients undergoing THA.

The current study is not without limitations. The retrospective design limits causal inference, and reliance on administrative coding may lead to inaccuracies in HTN and adverse event classification. Although matching and multivariate analyses were employed, selection biases and confounding factors could still affect results. In addition, because the administrative claims data do not provide blood pressure measurements or indicators of HTN control, we were unable to differentiate between controlled and uncontrolled HTN, which may represent clinically meaningful subgroups with differing risks.

In summary, this large-cohort study identified HTN to be diagnosed for more than half of patients undergoing THA. The clear correlation of HTN with many adverse outcomes and mildly (but statistically) lower 5-year implant survival highlights the need for further consideration of this variable. Although there could be other confounding factors that may not have been fully controlled, those with HTN are clearly an “at-risk” group.
